# Loss of endothelial glucocorticoid receptor accelerates organ fibrosis in *db/db* mice

**DOI:** 10.1152/ajprenal.00105.2023

**Published:** 2023-08-17

**Authors:** Swayam Prakash Srivastava, Julie E. Goodwin

**Affiliations:** ^1^Department of Pediatrics, Yale University School of Medicine, New Haven, Connecticut, United States; ^2^Vascular Biology and Therapeutics Program, Yale University School of Medicine, New Haven, Connecticut, United States; ^3^Department of Cellular and Molecular Physiology, Yale University School of Medicine, New Haven, Connecticut, United States

**Keywords:** diabetes, endothelium, fibrosis, steroids

## Abstract

Endothelial cells play a key role in maintaining homeostasis and are deranged in many disease processes, including fibrotic conditions. Absence of the endothelial glucocorticoid receptor (GR) has been shown to accelerate diabetic kidney fibrosis in part through upregulation of Wnt signaling. The *db/db* mouse model is a model of spontaneous type 2 diabetes that has been noted to develop fibrosis in multiple organs over time, including the kidneys. This study aimed to determine the effect of loss of endothelial GR on organ fibrosis in the *db/db* model. *db/db* mice lacking endothelial GR showed more severe fibrosis in multiple organs compared with endothelial GR-replete *db/db* mice. Organ fibrosis could be substantially improved either through administration of a Wnt inhibitor or metformin. IL-6 is a key cytokine driving the fibrosis phenotype and is mechanistically linked to Wnt signaling. The *db/db* model is an important tool to study the mechanisms of fibrosis and its phenotype in the absence of endothelial GR highlights the synergistic effects of Wnt signaling and inflammation in the pathogenesis or organ fibrosis.

**NEW & NOTEWORTHY** The major finding of this work is that endothelial glucocorticoid receptor-mediated upregulation of Wnt signaling and concurrent hyperinflammation work synergistically to exacerbate organ fibrosis in a genetic mouse model of diabetes. This study adds to our understanding of diabetic renal fibrosis and has important implications for the use of metformin in this condition.

## Graphical abstract

**Figure:**
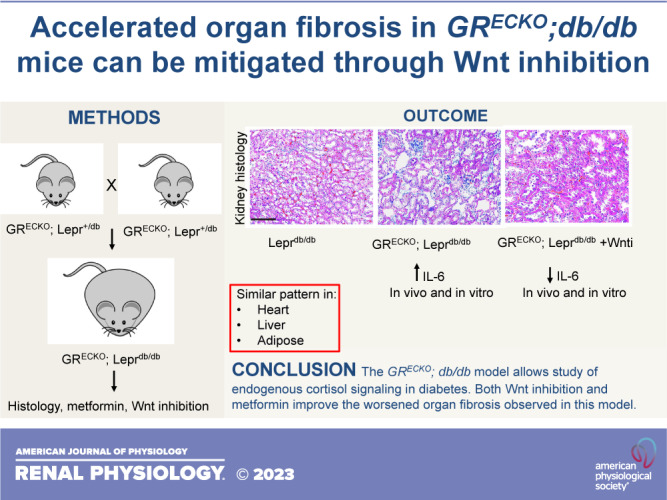

## INTRODUCTION

Despite the rising incidence of fibrotic kidney disease, driven to a great extent by a continued increase in the prevalence of diabetes (1, 2), molecular mechanisms of renal fibrosis remain incompletely understood. One important tool to study this phenomenon is the C57BL/6 *db/db* mouse model, which demonstrates type 2 diabetes, significant albuminuria, renal histological damage, and reduced renal function by 32 wk of age (3). Studies of *db/db* mice have demonstrated that these animals display deranged composition and morphology of key organs such as kidney, liver, heart, and adipose tissue, as well as significantly different viscoelastic properties, even when compared with the *ob/ob* mouse, which also possesses a leptin mutation (4), highlighting the fact that biomechanical properties may be involved in the pathogenesis of diabetic complications. Additional insights gained through the study of the *db/db* model have revealed other mechanistic underpinnings of diabetes including oxidative stress, advanced glycation end products, renin-angiotensin-aldosterone system (RAAS) activation, and unchecked inflammation (3).

Previously, we demonstrated that mice lacking the endothelial glucocorticoid receptor (*GR*^ECKO^) were highly susceptible to streptozotocin-induced diabetic nephropathy and demonstrated augmented Wnt signaling, aberrant cytokine reprogramming, and suppressed fatty acid oxidation (FAO) (5, 6), leading to the conclusion that the endothelial glucocorticoid receptor (GR) is a key regulatory molecule in the pathophysiology of renal fibrosis. Furthermore, we have also demonstrated the crucial role of endothelial cell-podocyte cross talk, mediated via GR, in influencing the diabetic nephropathy phenotype (7). Evolving understanding of the complicated Wnt signaling pathway suggests a favorable role in kidney repair when transiently activated, but a detrimental effect that promotes injury and fibrosis in states of unremitting Wnt activation (8).

In this work, we show that when bred onto the *db/db* background, *GR*^ECKO^ mice display widespread organ fibrosis; high-fat diet (HFD) feeding of *GR*^ECKO^ mice can produce a similar phenotype. This phenotype can be partially rescued by either administration of metformin or a small molecule Wnt inhibitor. Furthermore, IL-6 is a key cytokine driving fibrosis in this model. We conclude that both the hyperinflammatory state and the concurrent uncontrolled augmented Wnt signaling, which result from the loss of endothelial GR (6, 9, 10), are further exacerbated by either HFD feeding or the *db/db* background. The *db/db;GR*^ECKO^ model is a highly unique model, which illustrates the key importance of both inflammation and Wnt signaling in the pathophysiology of diabetic nephropathy.

## METHODS

### Animal Experimentation

All experiments were performed according to a protocol approved by the Institutional Animal Care and Use Committee at the Yale University School of Medicine and were in accordance with the National Institute of Health (NIH) guidelines for the care of laboratory animals. Mice were housed at an ambient temperature of 68–79°F with humidity that ranged between 30% and 70%. They were exposed to 12:12-h light/dark cycles. Mice lacking endothelial GR (*GR*^ECKO^) were successively bred to heterozygous *Lepr*^+/db^ mice to achieve the target mice: *db/db;GR*^ECKO^. Wnt inhibitor (LGK974) was provided to 16-wk-old male mice by gavage using a dose of 5 mg/kg at a frequency of six doses per week for 8 wk, as described previously (11). Metformin was provided at 100 mg/kg by gavage for 8 wk to some mice. In other experiments, mice were received a HFD containing 40% fat (Research Diets, D12108). For glucose tolerance testing (GTT), mice were fasted overnight. On the following day, blood glucose profiles were measured at 0 min (baseline) and then at 30, 60, 90, and 120 min after oral glucose load of 3 g/kg body wt. Aside from this overnight fast for GTT, all mice had free access to food and water during experiments. Blood was obtained by retro-orbital bleed during experiments. Blood glucose was measured by glucose strips. Tissues and blood were harvested at the time of euthanasia. Some tissues were minced and stored at −80°C for gene expression and protein analysis. Other tissues were placed immediately in optimal cutting temperature compound for frozen sections or 4% paraformaldehyde for histological staining.

### Morphological Evaluation

Masson’s trichrome-stained images were evaluated by ImageJ software, and the fibrotic areas were estimated. Deparaffinized sections were incubated with picrosirius red solution for 1 h at room temperature. The slides were washed twice with acetic acid solution for 30 s per wash. The slides were then dehydrated in absolute alcohol three times, cleared in xylene, and mounted with a synthetic resin. Sirius red staining was analyzed using ImageJ software, and fibrotic areas were quantified.

### In Vitro Experiments and siRNA Transfection

Human umbilical vein endothelial cells (HUVECs) were used at passages four to eight and cultured in Endothelial Basal Medium-2 media with growth factors and 10% serum. Human GR-specific siRNA (Invitrogen) was used at a concentration of 100 nM for 48 h to effectively knock down GR. Cells were treated with 10 μg/mL IgG control or IgG N-IL-6 (Bio X Cell), Wnt3a 200 ng/mL (PeproTech), or DKK-10 μg/mL (R&D Systems) according to the experimental plan. When the cells reached 70% confluence, conditioned media from control siRNA- and GR siRNA-transfected HUVECs were tested for IL-6 expression.

### RNA Isolation and qPCR

Total RNA was isolated using standard TRIzol protocol. RNA was reverse transcribed using the iScript cDNA Synthesis Kit (Bio-Rad) and qPCR was performed on a Bio-Rad C1000 Touch thermal cycler using the resultant cDNA, as well as qPCR Master Mix and gene-specific primers. Results were quantified using the delta-delta-cycle threshold (Ct) method (ΔΔCt). All experiments were performed in triplicate and 18S was used as an internal control. The following primers were used:

α-smooth muscle actin (α-SMA):

Forward 5′- CTGACAGAGGCACCACTGAA and reverse 5′- GAAATAGCCAAGCTCAG

Fibroblast-specific protein 1 (FSP-1):

Forward 5′- TTCCAGAAGGTGATGAG and reverse 5′- TCATGGCAATGCAGGACAGGAAGA

Axin2:

Forward 5′- AACCTATGCCCGTTTCCTCTA and reverse 5′- GAGTGTAAAGACTTGGTCCACC

18S:

Forward 5′- TTCCGATAACGAACGAGACTCT and reverse 5′- GGCTGAACGCCACTTGTC

All primers were synthesized by the Keck Oligonucleotide Synthesis facility at Yale School of Medicine.

### Cytokine Measurements

IL-1β, IL-6, and IFN-γ were measured by cytokine array using the Luminex platform.

### Statistical Significance

All values are expressed as means ± SE and were analyzed using the statistical package for GraphPad Prism 7 (GraphPad Software Inc., La Jolla, CA). Student’s *t* test was used to evaluate binary comparisons and one-way ANOVA, followed by Tukey’s test to analyze significance when comparing multiple independent groups. In each experiment, *n* represents the number of separate experiments (in vitro) or the number of mice (in vivo). Technical replicates were used to ensure the reliability of single values. The data were considered statistically significant at *P* < 0.05.

## RESULTS

### Loss of Endothelial GR Causes Organ Fibrosis in *db/db* Mice

**Figure 1.**
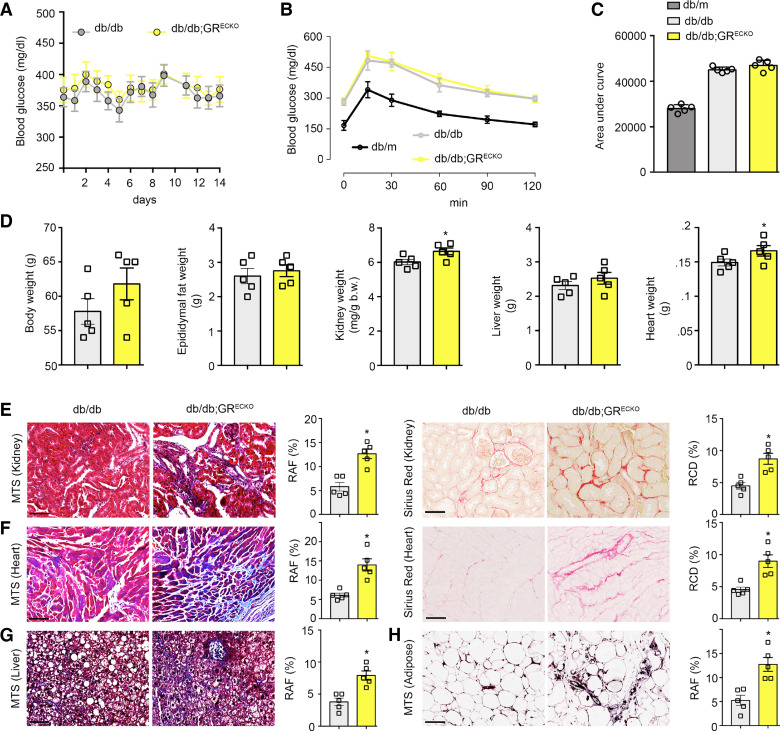
Loss of endothelial cell GR causes organ fibrosis in *db/db* mice. *A*: postprandial blood glucose measurements after 12-h fasts over a 2-wk period in *db/db* controls and endothelial cell-specific knockout GR in *db/db* mice (*db/db;GR*^ECKO^). *n* = 5/group. *B*: glucose tolerance test (GTT) in control *db/db* mice and in *db/db;GR*^ECKO^ mice. Heterozygous (nondiabetic) ± *db* (*db/m*) mice were included as a control. *n* = 5/group. *C*: AUC in control *db/db*, *db/m*, and *db/db;GR*^ECKO^ mice. *n* = 5/group. *D*: physiological parameters including body weight, epididymal fat weight, kidney weight/body weight, liver weight, and heart weight in control *db/db* and *db/db;GR*^ECKO^ mice. *n* = 5/group. *E*: MTS and sirius red staining analysis in the kidneys of control *db/db* and *db/db;GR*^ECKO^ mice. Representative images at ×30 magnification are shown. *n* = 5/group. Relative area of fibrosis (RAF) and relative collagen deposition (RCD) were quantified using ImageJ. *F*: MTS and sirius red staining analysis in heart sections of control *db/db* and *db/db;GR*^ECKO^ mice. Representative images at ×20 are shown. *n* = 5/group. *G* and *H*: MTS in liver (*G*) and adipose tissue (*H*) from control *db/db* and *db/db;GR*^ECKO^ mice. Representative pictures at ×20x are shown. *n* = 5/group. Scale bar: 50 μm. Data represent means ± SE. *P ≤ 0.05. One-way ANOVA with Tukey’s test was used for *B* and *C*. Student’s *t* test was used for *D*–*H*. AUC, area under the curve; GR, glucocorticoid receptor; MTS, Masson's trichrome stain.

We bred *GR*^ECKO^ mice onto the *db/db* background and assessed postprandial glucose measurements after a 12-h fast over a 2-wk period. Control *db/db* mice and *db/db;GR*^ECKO^ mice demonstrated similar serum glucose levels over a 14-day period (Fig. 1*A*) as well as similar values during an oral glucose tolerance test after a glucose load of 3 mg/g body wt (Fig. 1, *B* and *C*). Further phenotyping showed that control *db/db* and *db/db;GR*^ECKO^ mice had similar body weight, epididymal fat weight, and liver weight, but *db/db;GR*^ECKO^ mice had higher kidney weight/body weight ratios and increased heart weight (Fig. 1*D*). Histological examination of the kidneys and hearts from these animals clearly showed more fibrosis (Masson’s trichrome stain) and collagen deposition (sirius red) in organs from the *db/db;GR*^ECKO^ mice (Fig. 1, *E* and *F*). Interestingly, liver and adipose tissue from *db/db;GR*^ECKO^ mice also showed more fibrosis compared with *db/db* controls, even though there was no change detected in these organs’ weight (Fig. 1, *G* and *H*). Lower magnification images (×10) of these tissues demonstrate that the fibrosis is widespread in each case and are provided in Supplemental Fig. S1.

### Wnt Inhibition Improves Organ Fibrosis in Diabetes

**Figure 2.**
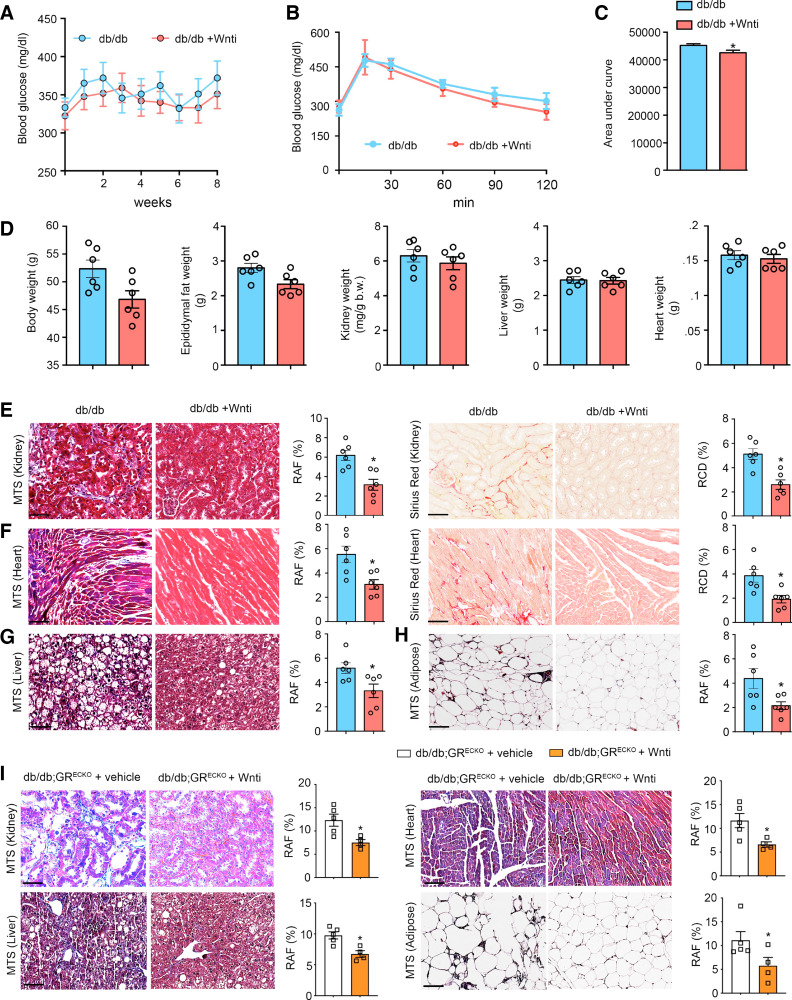
Pharmacological inhibition of Wnt signaling abolishes the fibrogenic phenotype in *db/db* mice. *A*: postprandial blood glucose measurements in the Wnt inhibitor-treated *db/db* mice and untreated control *db/db* mice. LGK-974 (Wnti) was administered at 5 mg/kg body wt orally by gavage. *B*: glucose tolerance test (GTT) analysis in the Wnti-treated *db/db* mice and untreated control *db/db* mice. *n* = 6/group. *C*: AUC for Wnti-treated *db/db* mice and untreated control *db/db* mice. *n* = 6/group. *D*: physiological parameters such as body weight, epididymal fat, kidney weight/body weight, liver weight, and heart weight in Wnti-treated *db/db* mice and untreated control *db/db* mice. *n* = 6/group. *E*: MTS and sirius red staining in the kidneys of Wnti-treated *db/db* mice and untreated control *db/db* mice. Representative images at ×30 are shown. *n* = 6/group. Relative area of fibrosis (RAF) and relative collagen deposition (RCD) were quantified using ImageJ. *F*: MTS and sirius red staining analysis in heart sections from Wnti-treated *db/db* mice and untreated control *db/db* mice. Representative images at ×20 are shown. *n* = 6/group. *G* and *H*: MTS in liver (*G*) and adipose tissue (*H*) from Wnti-treated *db/db* mice and untreated control *db/db* mice. *I*: MTS staining of representative kidney (×30), liver (×20), heart (×20), and adipose (×20) tissue sections from vehicle-treated or Wnti-treated *db/db;GR^ECKO^* mice. *n* = 5/group. Scale bar: 50 μm. Data represent the means ± SE. *P ≤ 0.05. Student’s *t* test was used for analysis of statistical significance. AUC, area under the curve; MTS, Masson’s trichrome stain.

Next, we tested the effect of Wnt inhibition on glycemic control in *db/db* mice using a small molecule Wnt inhibitor, LGK974 (Wnti). Postprandial glucose measurements after a 12-h fast in *db/db* mice that were either untreated or treated with Wnti for 8 wk revealed a trend toward improved glycemic control in treated mice (Fig. 2*A*). A formal oral glucose tolerance test did show a small but significant improvement in glucose control in the Wnti-treated mice, which was most pronounced at the later timepoints (Fig. 2, *B* and *C*). Additional phenotyping showed no differences between these two groups of mice (Fig. 2*D*). Histological examination of organs from untreated and Wnti-treated *db/db* mice showed improvement in organ fibrosis in the Wnti-treated group (Fig. 2, *E*–*H*). When *db/db;GR*^ECKO^ mice were treated with Wnti, improvement in the extent of organ fibrosis was also observed in all tissues studied as shown in Fig. 2*I*.

### Wnt Inhibition Is as Effective as Metformin in Preventing Organ Fibrosis in HFD-Fed Mice

**Figure 3.**
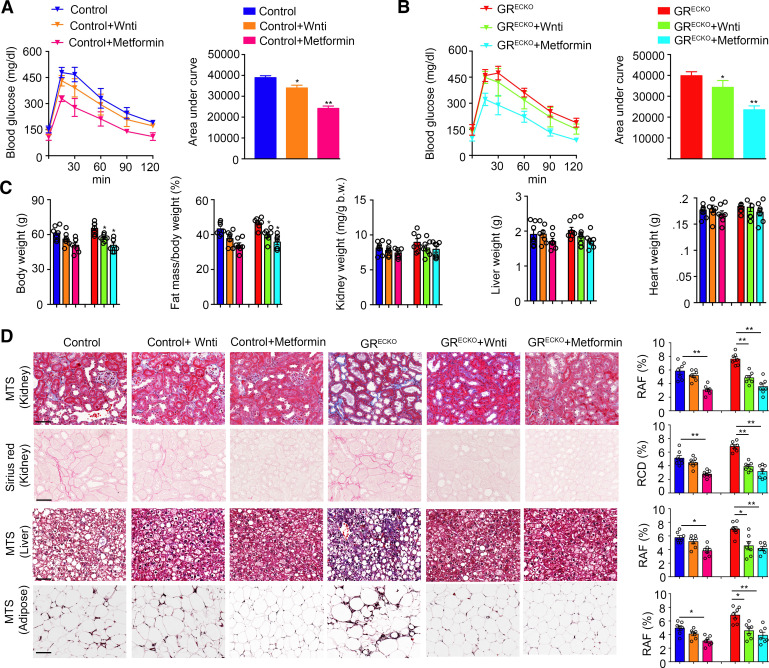
Effect of Wnti or metformin treatment in HFD-induced organ fibrosis. *A*: GTT and AUC in HFD-fed control mice either untreated, treated with Wnti, or treated with metformin. *n* = 7/group. *B*: GTT and AUC in HFD-fed *GR*^ECKO^ mice either untreated, treated with Wnti, or treated with metformin. *n* = 7/group. Wnti was administered at 5 mg/kg body wt and metformin was administered at 100 mg/kg body wt. *C*: physiological parameters such as body weight, fat mass/body weight, kidney weight/body weight, liver weight, and heart weight in the HFD-fed control or *GR*^ECKO^ mice either untreated, treated with Wnti, or treated with metformin. *n* = 7/group. *D*: MTS and sirius red staining from kidneys and heart sections. MTS staining from liver and adipose tissue is also shown. *n* = 7/group. Relative area of fibrosis (RAF) and relative collagen deposition (RCD) were quantified using ImageJ. Scale bar: 50 μm. Data represent means ± SE. *P ≤ 0.05 and **P ≤ 0.01. One-way ANOVA with Tukey’s test was used for analysis of statistical significance. AUC, area under the curve; GTT, glucose tolerance test; HFD, high-fat diet; MTS, Masson’s trichrome stain.

Prolonged HFD feeding leads to obesity, insulin resistance, and eventually diabetes. Based on our previous work demonstrating a striking benefit of Wnt inhibition on renal fibrosis in streptozotocin-induced diabetes (5), we assessed whether blockade of Wnt signaling was able to temper fibrosis in HFD-fed control (*GR*^fl/fl^ and *Cre^−^*) and *GR*^ECKO^ mice. Metformin, an oral antihyperglycemic agent that improves insulin sensitivity and fibrosis in diabetes, was used as a positive control. Mice that had been HFD fed for 20 wk were either untreated, treated with Wnti for 8 wk, or treated with metformin for 8 wk and then subjected to an oral glucose tolerance test. Interestingly, Wnt inhibition was able to partially blunt the hyperglycemic response, but not to the same extent as metformin (Fig. 3*A*). A similar result was achieved when HFD-fed *GR*^ECKO^ mice were exposed to the same treatments (Fig. 3*B*). HFD-fed *GR*^ECKO^ mice treated with either Wnti or metformin demonstrated lower body weight and decreased fat mass compared with similarly treated controls but no differences in organ weights were observed (Fig. 3*C*). Histology from the kidney, heart, liver, and adipose tissue from mice in each of the six treatment conditions was examined. Metformin, but not Wnti, was able to significantly improve fibrosis in all organs studied in the control animals, whereas in the *GR*^ECKO^ mice both Wnti and metformin were able to significantly improve organ fibrosis (Fig. 3*D*); the magnitude of the effect of metformin and Wnti in *GR*^ECKO^ mice was similar.

### IL-6 Is a Key Cytokine Driving the Fibrosis Phenotype In Vitro

**Figure 4.**
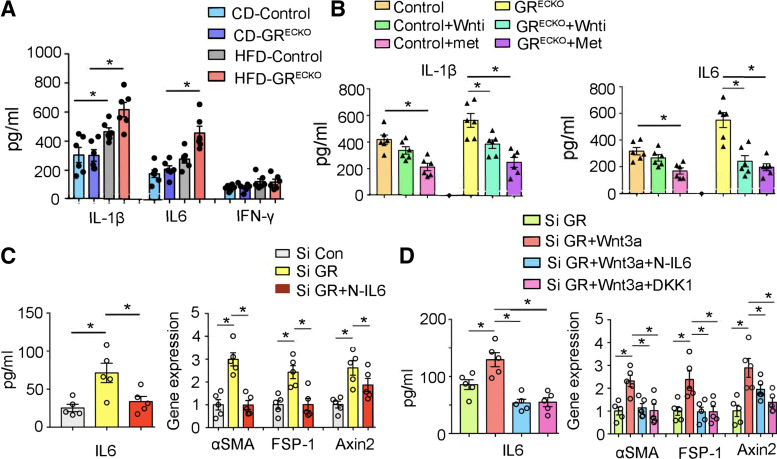
Both GR loss and Wnt activation are associated with proinflammatory and profibrotic signaling pathways. *A*: IL-1β, IL-6, and IFN-γ were measured in plasma from control diet (CD)- and HFD-fed control and *GR*^ECKO^ mice using Luminex cytokine array analysis. *n* = 6/group. *B*: IL-1β and IL-6 levels from plasma from HFD-fed control mice treated with Wnti, metformin, or untreated and HFD-fed *GR*^ECKO^ mice treated with Wnti, metformin, or untreated are shown. *n* = 6/group. *C*: IL-6 levels in the media of cultured HUVECs treated with either control siRNA (si Con), GR siRNA (si GR), or GR siRNA with neutralization antibody of IL-6 (si GR+N-IL-6) are shown. Relative expression of the indicated genes was normalized to 18S. *D*: IL-6 levels in conditioned media from GR siRNA-treated HUVECs treated with recombinant Wnt3a alone or in combination with either N-IL-6- or DKK-1-are shown. Relative expression of the indicated genes was normalized to 18S. Data represent means ± SE. *P < 0.05. One-way ANOVA with Tukey’s test was used for analysis of statistical significance. GR, glucocorticoid receptor; HFD, high-fat diet; HUVEC, human umbilical vein endothelial cell.

To investigate further the mechanism of fibrosis in these animals, we isolated plasma from control and *GR*^ECKO^ mice on both a standard chow diet and HFD and assessed the levels of key inflammatory cytokines including IL-1β, IL-6, and IFN-γ. HFD-fed animals of both genotypes demonstrated higher levels of IL-1β and IL-6, with HFD-fed *GR*^ECKO^ mice demonstrating the highest levels of all (Fig. 4*A*). There were no significant differences in IFN-γ levels. Next, plasma from control and *GR*^ECKO^ mice, untreated, treated with Wnti, or treated with metformin, was tested. IL-1β and IL-6 levels were again highest in *GR*^ECKO^ mice but could be suppressed by ∼50% with both Wnti treatment and metformin treatment, comparable to the levels of similarly treated controls (Fig. 4*B*). To examine this phenomenon in a cell culture system, HUVECs were treated with control siRNA alone, GR siRNA alone, or GR siRNA and an IL-6 neutralization antibody (N-IL-6). As shown in Fig. 4*C*, GR siRNA-treated cells had the highest levels of IL-6 and highest gene expression of the associated fibrogenic markers *α-SMA* and *FSP-1*, as well as the Wnt-dependent gene *axin2*; the expression of all of these was significantly suppressed with the administration of N-IL-6.

Finally, to determine whether the IL-6-mediated fibrotic effects were mechanistically tied to Wnt signaling, the GR siRNA-treated cells were either left untreated or concurrently treated with the canonical Wnt ligand Wnt3a (alone), Wnt3a and N-IL-6, or Wnt3a and DKK-1, an inhibitor of canonical Wnt. Administration of Wnt3a alone significantly increased IL-6 levels, which could be similarly suppressed with either N-IL-6 or DKK-1. The mRNA expression of fibrogenic markers and *axin2* was significantly increased by Wnt3a and could be similarly suppressed by either N-IL-6 or DKK-1, suggesting that IL-6 expression is directly linked to Wnt signaling.

## DISCUSSION

The major finding of this work is that endothelial GR-mediated upregulation of Wnt signaling and concurrent hyperinflammation work synergistically to exacerbate organ fibrosis in a model of diabetes. This phenomenon is analogous to the “two-hit” hypothesis that is observed in other conditions with renal manifestations such as autosomal-dominant polycystic kidney disease (ADPKD) (12), tuberous sclerosis (13), and von Hippel-Lindau disease (14). Recent work also demonstrates that nonalcoholic fatty liver disease (NAFLD), the risk of which is heightened by the presence of type 2 diabetes, is characterized by a “two-hit” or even a “multihit” hypothesis, which also includes a hyperinflammatory state and dysregulated Wnt signaling in addition to triglyceride accumulation in hepatocytes (15).

Metformin is an old and inexpensive drug that remains first-line therapy for type 2 diabetes mellitus. However, recognition of its pleiotropic effects has suggested that it may be beneficial in a wide variety of chronic diseases. For example, data from both animals and humans support the notion that metformin can modulate inflammation (16). Metformin is able to shift the balance of macrophages in favor of the M2 subtype, which decreases the release of cytokines such as TNF-α, IL-β, and IL-6, and heightens the anti-inflammatory response (17). Metformin was recently shown to suppress high-fat diet-induced upregulated Wnt signaling in a mouse model of age-related leaky gut and inflammation (18).

With regard to use in kidney disease, metformin has been shown to be associated with suppression of oxidative stress and inflammation in a rat model of diabetic nephropathy (19). In this study, the authors demonstrated that these improvements were specifically mediated by the inhibition of renal artery angiotensin II receptor type I/endothelin-1 axis (19), suggesting that much of the damage in diabetic nephropathy in this model was mediated via vascular effects. A beneficial effect of metformin on the progression of ADPKD was recently shown by Pastor-Soler et al., particularly in the reduction of key inflammatory markers and cell proliferation markers (20). One of the major limitations to broader use of metformin as a therapy for fibrosis is that the doses required often lead to clinically significant hypoglycemia. Indeed, in our study we did observe a 30–40% decrease in peak glucose concentration after glucose tolerance testing in HFD-fed, metformin-treated mice compared with controls. One potential solution to this problem is the development of a targeted drug delivery systems that involves the synthesis of nanoparticles composed of grafted chitosan, which acts as a metformin carrier and can be specifically delivered to renal tubular cells by megalin-mediated endocytosis (21).

A limitation of this study is that only male *db/db* mice were studied. There is literature to support the sex-specific effect of metformin (22). For example, metformin is generally associated with some degree of cardioprotection in women but not necessarily in men (23, 24). Conversely, metformin has been shown to prevent and reverse neuropathic pain only in male mice (25). It is unclear whether the effects of metformin in female *db/db;GR*^ECKO^ would have been the same as those observed in the male mice. Another limitation of the current work is that our mechanistic insights are derived mainly from in vitro studies, which cannot accurately mimic the pathophysiology of interstitial fibrosis. Future studies are planned to investigate fibrotic pathways in vivo in this model.

In conclusion, the *db/db;GR*^ECKO^ mouse model is a unique model in which the contributions of diabetes and endogenous cortisol signaling can be studied simultaneously. There is much we can learn regarding how the tonic anti-inflammatory effects of endothelial cortisol act as a “brake” on organ fibrosis and unchecked vascular inflammation.

## DATA AVAILABILITY

Data will be made available upon reasonable request.

## SUPPLEMENTAL DATA

10.6084/m9.figshare.23740122.v1 Supplemental Fig. S1: https://doi.org/10.6084/m9.figshare.23740122.v1.

## GRANTS

This work was supported by National Institutes of Health (NIH) Grant HL131952 (to J.E.G.).

## DISCLOSURES

No conflicts of interest, financial or otherwise, are declared by the authors.

## AUTHOR CONTRIBUTIONS

S.P.S. conceived and designed research; S.P.S. performed experiments; S.P.S. and J.E.G. analyzed data; J.E.G. interpreted results of experiments; S.P.S. prepared figures; J.E.G. drafted manuscript; J.E.G. edited and revised manuscript.
